# Cytolytic Activity of CAR T Cells and Maintenance of Their CD4+ Subset Is Critical for Optimal Antitumor Activity in Preclinical Solid Tumor Models

**DOI:** 10.3390/cancers13174301

**Published:** 2021-08-26

**Authors:** Marianna Csaplár, János Szöllősi, Stephen Gottschalk, György Vereb, Árpád Szöőr

**Affiliations:** 1 Department of Biophysics and Cell Biology, Faculty of Medicine, University of Debrecen, 4032 Debrecen, Hungary; csaplar.marianna@med.unideb.hu (M.C.); szollo@med.unideb.hu (J.S.); 2 MTA-DE Cell Biology and Signaling Research Group, Faculty of Medicine, University of Debrecen, 4032 Debrecen, Hungary; 3 Department of Bone Marrow Transplantation and Cellular Therapy, St. Jude Children’s Research Hospital, Memphis, TN 38105, USA; stephen.gottschalk@stjude.org; 4 Faculty of Pharmacy, University of Debrecen, 4032 Debrecen, Hungary

**Keywords:** solid tumor, chimeric antigen receptor (CAR), immunotherapy, cell therapy, T cell expansion, T cell persistence, HER2-positive tumors, CD4+, CD8+

## Abstract

**Simple Summary:**

Adoptively transferred T cells expressing recombinant chimeric antigen receptors (CAR T cells) have been approved for the therapy of hematological malignancies of the B cell lineage. However, CAR T cell therapy for patients with solid tumors so far has shown limited benefits. Correlative clinical studies of patients with hematological malignancies have suggested that less differentiated CAR T cells have improved anti-leukemic activity. We have therefore investigated the role of differentiation on the anti-tumor activity of CAR T cells targeting the solid tumor antigen HER2 in preclinical models. We utilized different activation/expansion protocols, and explored whether different co-stimulatory domains in the CAR construct influence the short- and long-term efficacy of HER2-CAR T cells. We demonstrate that the CAR T cell product with the highest proportion of effector cells and maintaining a good balance of CD4+/CD8+ cells is the most effective against solid tumors both in vitro and in vivo.

**Abstract:**

Correlative clinical studies of hematological malignancies have implicated that less differentiated, CD8+-dominant CAR T cell products have greater antitumor activity. Here, we have investigated whether the differentiation status of CAR T cell products affects their antitumor activity in preclinical models of solid tumors. We explored if different activation/expansion protocols, as well as different co-stimulatory domains in the CAR construct, influence the short- and long-term efficacy of CAR T cells against HER2-positive tumors. We generated T cell products that range from the most differentiated (CD28.z; OKT3-antiCD28/RPMI expansion) to the least differentiated (41BB.z; OKT3-RetroNectin/LymphoONE expansion), as judged by cell surface expression of the differentiation markers CCR7 and CD45RA. While the effect of differentiation status was variable with regard to antigen-specific cytokine production, the most differentiated CD28.z CAR T cell products, which were enriched in effector memory T cells, had the greatest target-specific cytolytic activity in vitro. These products also had a greater proliferative capacity and maintained CD4+ T cells upon repeated stimulation in vitro. In vivo, differentiated CD28.z CAR T cells also had the greatest antitumor activity, resulting in complete response. Our results highlight that it is critical to optimize CAR T cell production and that optimal product characteristics might depend on the targeted antigen and/or cancer.

## 1. Introduction

Genetically modifying T cells to express synthetic chimeric antigen receptors (CARs) [1] is one of the promising strategies for the immunotherapy of cancer. Since 2017, the US Food and Drug Administration (FDA) has approved five successful therapies for the treatment of hematological malignancies, including childhood acute B cell lymphoblastic leukemia (tisagenlecleucel) [2], diffuse large B cell lymphoma (axicabtagene ciloleucel and lisocabtagene maraleucel) [3,4], relapsed/refractory mantle cell lymphoma (brexucabtagene autoleucel) [5], and myeloma multiplex (idecabtagene vicleucel) [6]. Clinically, development of these CAR T cell products has represented a paradigm shift in the treatment of chemotherapy-resistant leukemias and lymphomas. However, the clinical experience with CAR T cells for solid tumors has been disappointing [7].

While the response rate of CAR T cells against hematological malignancies is impressive, limited in vivo persistence has emerged as a major limitation, resulting in relapse [8]. Numerous approaches have been developed to overcome this challenge, including optimizing CAR constructs, evaluating different methods for in vitro culturing, and exploring different CD4+/CD8+ and naïve/central memory/effector memory/terminal effector subset compositions [9]. It was also demonstrated that inclusion of 41BB as opposed to CD28 in the CAR architecture promoted the outgrowth of CD8+ central memory T cells [10,11]. In pre-clinical and clinical studies for hematological malignancies, less differentiated CD19-CAR T cell products, enriched in naïve and central memory T cells, exhibited superior anti-tumor activity and long-term persistence in vivo [12,13,14]. Moreover, naïve and central memory T cell-derived CD8+ and CD4+ CAR T cells performed better than those derived from effector memory T cells [15]. However, there is no experimental evidence as to whether these findings also apply to solid tumors.

Recently, RetroNectin, a recombinant human fibronectin fragment that is widely used to enhance gene transfer with retroviral vectors, has emerged as a promising stimulator of T cell proliferation. In combination with the T cell-activating anti-CD3 monoclonal antibody, OKT3, RetroNectin enhanced T cell expansion while preserving a naïve and central memory phenotype [16,17,18]. Furthermore, RetroNectin influenced the CD4+ and CD8+ composition of T cell products by inhibiting apoptosis of CD8+ T cells, shifting the cell composition towards a cytolytic phenotype over the time of in vitro culturing [17] as well as during in vivo persistence [19]. However, despite these encouraging results, at present, OKT3-RetroNectin activation has not been compared to standard OKT3-antiCD28 activation in the context of CAR T cell therapy for solid tumors. In addition, the influence of CD28 or 41BB costimulatory domains within the CAR architecture has not been explored.

We therefore generated HER2.CD28.z and HER2.41BB.z CAR T cells by using a standard OKT3-antiCD28 stimulation protocol, followed by expansion in complete RPMI culture media supplemented with IL-7 and IL-15 (*OKT3-antiCD28/RPMI*), or the above-mentioned OKT3-RetroNectin stimulation protocol followed by expansion in LymphoONE culture media supplemented with IL-7 and IL-15 (*OKT3-RetroNectin/LymphoONE*), a culture media that was specifically formulated to restrain human T cell differentiation during their expansion [20]. Using these two stimulation/culture conditions and CD28.z and 41BB.z CARs, we were able to create a set of cell products that ranged from differentiated (CD28.z CAR T cells manufactured with *OKT3-antiCD28/RPMI*) to least differentiated (41BB.z CAR T cells manufactured RetroNectin/LymphoONE), as judged by cell surface expression of the differentiation markers CCR7 and CD45RA. Our detailed functional analysis demonstrates that effector memory-enriched CD28.z CAR T cell products have superior effector functions in vitro and in vivo. Thus, our results highlight that optimal CAR T cell product characteristics might depend on the targeted antigen and/or cancer.

## 2. Materials and Methods

All materials were purchased from Sigma-Aldrich (St. Louis, MO, USA) unless indicated otherwise.

### 2.1. Cells and Culture Conditions

HEK 293T packaging cells, the triple-negative human breast cancer cell line MDA-MB-468, and N87 human gastric carcinoma cell lines were purchased from the American Type Culture Collection (ATCC, Manassas, VA, USA). The generation of MDA-MB-468 stably expressing HER2 (MDA-HER2) was previously described [21]. These cells were cultured in Dulbecco’s Modified Eagle Medium (DMEM) supplemented with 2 mM GlutaMAX, 10% Fetal Bovine Serum (FBS), and antibiotics. The JIMT-1 human breast cancer cell line was established in the laboratory of Cancer Biology, University of Tampere, Finland [22]. These cells were cultured in a 1:1 ratio of Ham’s F-12 and DMEM supplemented with 20% FBS, 300 U/L insulin, 2 mmol/L GlutaMAX, and antibiotics. Primary human T cells and CAR T cells were cultured in either RPMI (Roswell Park Memorial Institute, Buffalo, NY, USA) medium supplemented with 2 mmol/L GlutaMAX, 10% FBS, and antibiotics, or LymphoONE T-Cell Expansion Xeno-Free Medium (Takara Bio, Kusatsu, Japan) supplemented with 2 mM GlutaMAX, 10% FBS, and antibiotics.

MDA.ffLuc, MDA-HER2.ffLuc, N87.ffLuc, and JIMT-1.ffLuc were generated by single cell cloning of MDA-MB-468, MDA-HER2, N87, and JIMT-1 cell lines following their transduction with a retrovirus encoding eGFP.ffLuc to express an enhanced green fluorescent protein (eGFP) and firefly luciferase (ffLuc) fusion gene.

In ADCC experiments, MDA, MDA-HER2, N87, and JIMT-1 cells were cultured in either complete RPMI medium or complete LymphoONE T-Cell Expansion Xeno-Free Medium.

All the cells and cell lines listed above were maintained in a humidified atmosphere containing 5% CO_2_ at 37 °C and were routinely checked for the absence of mycoplasma contamination.

### 2.2. Retrovirus Production and Transduction of T Cells

Retroviral particles were generated by transient transfection of HEK 293T cells with the HER2-CAR-encoding pSFG retroviral vectors [21,23], Peg-Pam-e plasmid containing the sequence for MoMLV gag-pol, and pMax.RD114 plasmid containing the sequence for RD114, using jetPrime transfection reagent (Polyplus, Illkirch, France). The backbones of the different HER2-specific chimeric antigen receptors consisted of the IgG heavy-chain signal peptide, the HER2-specific single-chain variable fragment FRP5, the IgG1 short hinge, a transmembrane region of human CD28 with CD28 or 41BB intracellular costimulatory endodomains (HER2.CD28.z and HER2.41BB.z, respectively), and the cytoplasmic region of human CD3 zeta [21,23]. Supernatants containing the retrovirus were collected after 48 h.

To generate HER2-CAR T cells, human peripheral blood mononuclear cells (PBMCs) were isolated by Ficoll gradient centrifugation. T cells (0.5 × 10^6^/mL) were stimulated in non-tissue culture 24-well plates precoated with 1 µg/mL anti-CD3e (clone: OKT3; Thermo Fischer, Waltham, MA, USA) and anti-CD28 (R&D Systems, Minneapolis, MN, USA) antibodies in 2 mL of complete RPMI (*OKT3-antiCD28/RPMI protocol*) or with 1 µg/mL anti-CD3e (clone: OKT3; Thermo Fischer, Waltham, MA, USA) antibody and 20 μg/mL of RetroNectin (Takara Bio, Kusatsu, Japan) in 2 mL of complete LymphoONE T-Cell Expansion Xeno-Free Medium (*OKT3-RetroNectin/LymphoONE protocol*). On day 2, human interleukin-7 (IL-7; 10 ng/mL) and human interleukin-15 (IL-15; 5 ng/mL) (Miltenyi Biotec, Bergisch Gladbach, Germany) were added to the cultures. T cells were transduced with retroviral particles on 20 μg/mL RetroNectin-coated plates on day 3 in the presence of IL-7 (10 ng/mL) and IL-15 (5 ng/mL). The expansion of T cells was subsequently supported with IL-7 and IL-15. OKT3/CD28 and OKT3/RetroNectin-activated non-transduced (NT) T cells were expanded in complete RPMI or LymphoONE medium supplemented with 2 mM GlutaMAX, 10% FBS, antibiotics, IL-7, and IL-15 using the same protocol. Following a 48 h incubation, cells were used for further experiments.

### 2.3. Flow Cytometry

HER2-CAR expression was confirmed using a HER2-Fc fusion protein (R&D Systems, Minneapolis, MN, USA) followed by Alexa Fluor 488-conjugated anti-human IgG (Invitrogen/Thermo Fisher/, Carlsbad, CA, USA) staining. T cell purity was determined by Alexa Fluor 647-conjugated anti-human CD3 antibody (BD Biosciences, San Jose, CA, USA) staining. In rechallenge assays, CD4 and CD8 positivity was tested by labeling with FITC-conjugated anti-CD4 (BD Biosciences, San Jose, CA, USA) and Alexa Fluor 647-conjugated anti-CD8 (YTC 182.20 [24,25]; prepared from hybridoma supernatant) antibodies. Memory phenotypes of the various CAR T cell populations were determined by their CCR7 (FITC Mouse anti-human CD197 (CCR7) Clone 150503; BD Biosciences, San Jose, CA, USA) and CD45RA (APC Mouse anti-human CD45RA Clone HI100; BD Biosciences, San Jose, CA, USA) expression. We identified naïve cells as CCR7 and CD45RA double-positive, central memory as CCR7-positive and CD45RA-negative, effector memory as CCR7 and CD45RA double-negative, and terminal effector T cells as CCR7-negative and CD45RA-positive. All markers used for flow cytometric analysis (antibodies and HER2-Fc recombinant protein) were applied at a 10 µg/mL final concentration for 10 min on ice. Analysis was performed on at least 10,000 cells per sample using a NovoCyte flow cytometer and NovoExpress software (ACEA Biosciences, San Diego, CA, USA).

### 2.4. In Vitro Rechallenge Assays

HER2.CD28.z and HER2.41BB.z CAR T cells were manufactured from *OKT3-antiCD28/RPMI-* and *OKT3-RetroNectin/LymphoONE*-stimulated PBMCs derived from four donors. Then, CAR T cell products were placed onto 1 µg/mL HER2-Fc (R&D Systems, Minneapolis, MN, USA) precoated plates. Every 3.5 days, effector cell number was determined by flow cytometry. At the same time, we calculated the proliferation rate by dividing the total effector cell number on the present day by the effector cells plated in the beginning of the last 3.5-day round. If the proliferation rate fell below 1, the available maximum amount of effector cells was placed onto fresh plates at the beginning of a new round. The experiment was concluded for a subset if the proliferation rate of the effector cells fell under 0.45.

At days 0, 3.5 and 10.5 of culturing, we analyzed the CD8/CD4 ratio and the memory phenotypes of the persisting CAR T cells by flow cytometry. Non-transduced (NT) T cells served as controls.

### 2.5. Cytokine Secretion Assay

Two-hundred thousand HER2.CD28.z and HER2.41BB.z CAR T cells prepared by using *OKT3-antiCD28/RPMI* and *OKT3-RetroNectin/LymphoONE* pre-stimulation and cell culture protocols were plated onto 1 µg/mL HER2-Fc precoated plates or cocultured with MDA-HER2, N87, or JIMT-1 HER2+ target cells at a 1:1 ratio. Following 24 h of culture, supernatant was harvested and analyzed for the presence of interferon (IFN) γ, interleukin (IL) 2, and secretable TIM-3 by ELISA or the Proteome Profiler Human XL Cytokine Array (both R&D systems, Minneapolis, MN, USA), according to the manufacturer’s instructions using a Synergy HT ELISA reader (BioTec, Winooski, VE, USA) or a FluorChem Q system (ProteinSimple, San Jose, CA, USA) for readout, respectively. Empty media, NT T cells, HER2+ target cells cultured without the presence of effector cells, and the HER2– MDA-MB-468 cell line served as controls.

### 2.6. Cytotoxicity Assay

Cytotoxic activity of CAR T cells against targets was determined by a luciferase-based cytotoxicity assay. MDA-MB-468, MDA-HER2, JIMT-1, and N87 cells expressing eGFP/ffLuc were plated in 96-well flat-bottom plates at a density of 10^5^ cells/well in triplicates. After 4 h, *OKT3-antiCD28/RPMI*- or *OKT3-RetroNectin/LymphoONE*-stimulated effector CAR T cells were added at a 1:1 effector to tumor cell ratio. Wells without effector cells served as untreated controls. After 24 h, luciferase activity was determined using a luciferase assay kit according to the manufacturer’s instructions (Promega, Madison, WI, USA) and a Synergy HT luminometer (BioTek, Winooski, VE, USA). Empty media, NT T cells, HER2+ target cells cultured without the presence of effector cells, and the HER2– MDA-MB-468.ffLuc cell line served as controls.

### 2.7. Phospho-Proteome Array

The Proteome Profiler Human Phospho-Kinase Array kit (R&D Systems, Minneapolis, MN, USA) was used for the parallel determination of the relative phosphorylation level of relevant protein kinases and substrates. First, we incubated 3.33 × 10^6^ CAR T cells in 1 mL culture medium from each subgroup (CM-enriched or EM-enriched, CD28.z or 41BB.z) on 1 µg/mL HER2-Fc precoated plates for 0.5, 2.5, or 7 min at 37 °C. Then, cell samples were pooled and washed with ice-cold PBS. Finally, 1 × 10^6^ cells were centrifuged at 1200 rpm for 5 min at 4 °C and lysed according to the manufacturer’s instructions. Cell lysates were incubated overnight at 4 °C on a rocking platform shaker on nitrocellulose membranes spotted in duplicate with capture antibodies. After three washings, the membranes were incubated with a cocktail of biotinylated detection antibodies for 2 h at room temperature, washed three times, and incubated with streptavidin-HRP for 30 min at room temperature. Finally, membranes were washed, visualized using the provided chemiluminescent detection reagents, and imaged with a FluorChem Q system (ProteinSimple, San Jose, CA, USA). Immunoreactive spot intensities were quantified using the software ImageJ/Fiji [26].

### 2.8. Cluster Analysis

Unsupervised two-way hierarchical clustering was performed on the average phospho-kinase levels using the R-based ClustVis [27]. Phosphoproteins that gave signals in all four samples below the negative control were not analyzed. Rows were centered, and unit variance scaling was applied to rows. Rows and columns were clustered using correlation distance and average linkage and displayed on a heat-map.

### 2.9. Xenograft Tumors and In Vivo Car T Cell Treatments

NSG (NOD.Cg-Prkdcscid/Il2rgtm1Wjl/SzJ) mice were purchased from The Jackson Laboratory and housed in a specific-pathogen-free environment. For testing the in vivo effector functions of the *OKT3-antiCD28/RPMI-* and *OKT3-RetroNectin/LymphoONE*-stimulated HER2.CD28.z and HER2.41BB.z CAR T cells, seven-week-old female NSG mice were administered a subcutaneous (s.c.) injection in both flanks, each containing 3 × 10^6^ JIMT-1.ffLuc cells in 100 µL of PBS mixed with an equal volume of Matrigel (BD Biosciences, San Jose, CA, USA). Tumor growth was monitored with an IVIS Spectrum CT instrument (Perkin Elmer, Waltham, MA, USA). Before measurement, isoflurane-anesthetized animals were injected IP with D-luciferin (150 mg/kg). A bioluminescence image was obtained and analyzed after 10 min using Living Image software Version 4.0 (Caliper Life Sciences, Waltham, MA, USA). A region of interest of the same size was drawn over the tumor region, and the intensity of the signal measured as total photons per second per square centimeter per steradian (p/s/cm^2^/sr) was obtained. Mice received a single dose of 2.5 × 10^6^ HER2-CAR or unmodified T cells i.v. on day 14 post-tumor cell inoculation. To monitor the in vivo persistence of HER2.CD28.z and HER2.41BB.z CAR T cells, we labeled the effectors with firefly luciferase.

### 2.10. Statistical Analysis

GraphPad Prism 5 software (GraphPad software, Inc., La Jolla, CA, USA) was used for statistical analysis. Data were presented as mean ± SD or SEM. For comparison between two groups, a two-tailed *t* test was used. For comparisons of three or more groups, one-way ANOVA with Bonferroni’s post hoc test was used. For the mouse experiments, survival, determined from the time of tumor cell injection, was analyzed by the Kaplan-Meier method and log-rank test. *p*-values < 0.05 were considered statistically significant.

## 3. Results

### 3.1. The OKT3-RetroNectin/LymphoONE CAR T Cell Generation Protocol Restrains T Cell Differentiation

**Figure 1:**
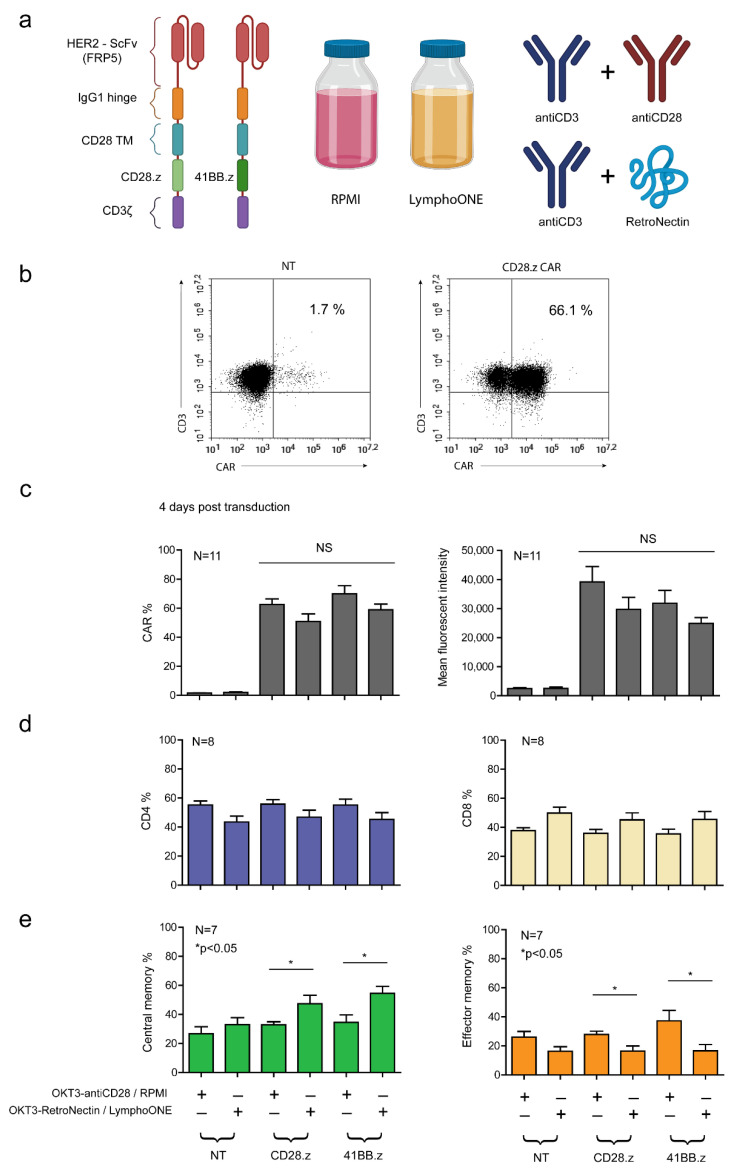
Generation of FRP5-derived HER2-CAR T cells with the two distinct cell culturing and two distinct stimulating methods, and assessing their distribution of CD4/CD8 and memory phenotypes. (**a**) Schematic diagram illustrating the modular composition of the CD28.z and 41BB.z HER2-CARs, the two media (RPMI and LymphoONE), and the two stimulation methods (antiCD3+antiCD28 and antiCD3+RetroNectin) applied in the experimental approach. (**b**) Representative flow cytometry dot-plot of CD28.z HER2-CAR T cells and non-transduced T cells. (**c**) HER2-CAR expression quantified using a HER2-Fc fusion protein followed by Alexa Fluor 647-conjugated anti-human IgG staining (*n* = 11). (**d**) CD4 and CD8 positivity tested by labeling with FITC-conjugated CD4 and Alexa Fluor 647-conjugated CD8 4 days post-transduction (*n* = 8). (**e**) Naïve (CCR7, CD45RA double-positive), central memory (CCR7-positive, CD45RA-negative), effector memory (CCR7, CD45RA double-negative), and terminal effector (CCR7-negative, CD45RA-positive) phenotype determined by labeling with FITC-conjugated anti-human CCR7 and APC-conjugated anti-human CD45RA 4 days post-transduction (*n* = 7). Histograms of the naïve and terminal effector phenotype frequency are provided in Supplementary Figure S2. Histograms represent the mean ± SD (*OKT3-antiCD28/RPMI* vs. *OKT3-RetroNectin/LymphoONE*: * *p* < 0.05).

To compare the role of various stimulation and cell culture protocols on the activation and anti-tumor function of CAR T cells, we generated primary human T cells that express a CAR with an FRP5 scFv-based HER2-specific recognition domain, an IgG1 short hinge, a CD28 transmembrane domain, and CD28.CD3ζ (HER2.CD28.z) or 41BB.CD3ζ (HER2.41BB.z) costimulatory and effector endodomains (Figure 1a). In our pipeline, PBMCs were first activated and then transduced with the CAR-encoding retroviral vectors. We assessed two stimulation and culture conditions: (1) anti-CD3 (OKT3) and anti-CD28 stimulation and complete RPMI culture media supplemented with IL-7 and IL-15 (*OKT3-antiCD28/RPMI*) [28], and (2) OKT3 and RetroNectin stimulation and LymphoONE culture media supplemented with IL-7 and IL-15 (*OKT3-RetroNectin/LymphoONE*) [18] (Figure 1a).

Mean transduction efficiency was determined by flow cytometry on day 4 post-transduction. We found that both HER2.CD28.z and HER2.41BB.z CARs were stably expressed on T cells, and that the *OKT3-antiCD28/RPMI* stimulation protocol induced higher—albeit non-significantly higher—transduction efficiencies (Figure 1b,c). The median expression was 62.6% and 69.9% for *OKT3-antiCD28/RPMI*-stimulated HER2.CD28.z and HER2.CD28.z CAR T cells, while *OKT3-RetroNectin/LymphoONE* stimulation resulted in 50.8% and 59% CAR positivity (Figure 1b,c). CAR T cells generated with the *OKT3-antiCD28/RPMI* protocol also expressed a greater number of CARs on their cell surface in comparison to CAR T cells generated with the *OKT3-RetroNectin/LymphoONE* protocol, as judged by mean fluorescent intensity (MFI) values (Figure 1c).

To assess the influence of different activation protocols on the development of different T cell subpopulations, we analyzed the effector and memory phenotype markers on day 4 post-transduction. CD8 and CD4 markers determined cytotoxic and helper T cell subsets, while CD45RA and CCR7 markers were used for evaluation of the differentiation status. Naïve-like cells (naïve) were defined as CD45RA+CCR7+, central memory-like cells (CM) as CD45RA–CCR7+, effector memory-like cells (EM) as CD45RA-CCR7-, and terminal effector-like cells (TE) as CD45RA+CCR7- T cells (Supplementary Figure S1). Our analysis revealed that CAR T cells generated with the *OKT3-RetroNectin/LymphoONE* protocol were slightly enriched in CD8+ cytotoxic lymphocytes (CD8% is 56.5% in NT, 53.2% in CD28.z, and 54.7% in 41BB.z, Figure 1d). CAR T cell products generated with the *OKT3-RetroNectin/LymphoONE* protocol had a higher percentage of CM cells in both CD28.z (*OKT3-antiCD28/RPMI* vs. *OKT3-RetroNectin/LymphoONE*: 32.8% vs. 47.3%, *p* = 0.049) and 41BB.z CAR T cell products (*OKT3-antiCD28/RPMI* vs. *OKT3-RetroNectin/LymphoONE*: 34.5% vs. 54.5%, *p* = 0.014). In contrast, CAR T cell products generated with the OKT3-antiCD28/RPMI protocol had a higher frequency of effector memory phenotypes (EM CD28.z: *OKT3-antiCD28/RPMI* vs. *OKT3-RetroNectin/LymphoONE*: 27.9% vs. 16.4%, *p* = 0.023; EM 41BB.z: *OKT3-antiCD28/RPMI* vs. *OKT3-RetroNectin/LymphoONE*: 37.3% vs. 16.6%, *p* = 0.029). However, neither activation/culture protocols had a significant influence on the frequency of naïve and terminal effector CAR T cells.

Likewise, we demonstrated that generating CAR T cells in *OKT3-antiCD28/LymphoONE* or *OKT3-RetroNectin/RPMI* results in intermediary phenotypes, which are not significantly different from each other or the *OKT3-antiCD28/RPMI* and *OKT3-RetroNectin/LymphoONE* protocols (Supplementary Figure S2). Thus, we decided to compare these latter two conditions, which yielded significantly different phenotypes in further experiments.

### 3.2. The Effects of HER2-CAR T Cell Phenotype on Cytokine Production Post-Antigen-Specific Activation Depends on the Utilized Costimulatory Domain and the Method of CAR Activation

**Figure 2:**
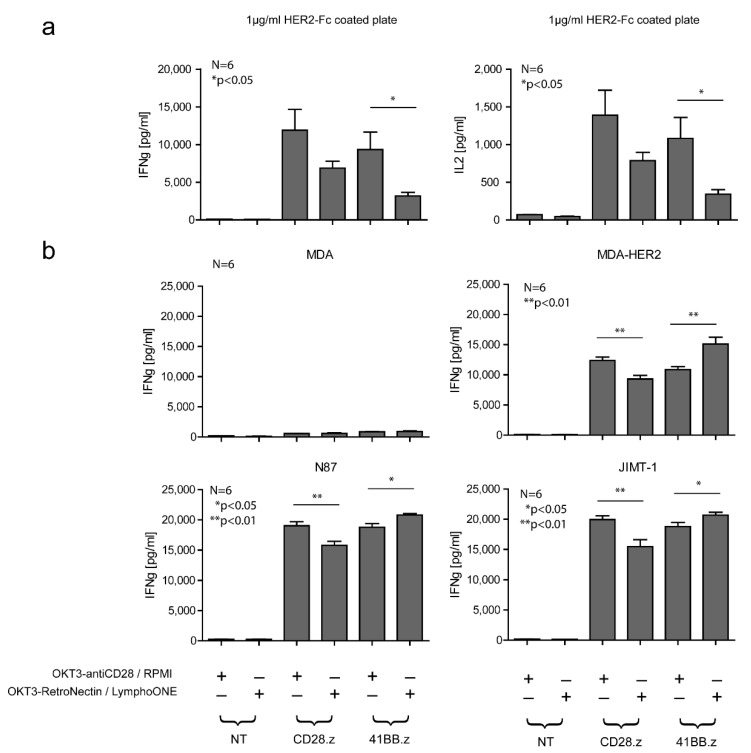
Cytokine release of HER2-CAR T cells prepared with the two different stimulation protocols. (**a**) 2 × 10^5^ CD28.z and 41BB.z HER2-CAR T cells prepared with the two different stimulation and cell culture protocols were plated onto 1 µg/mL HER2-Fc precoated plates or (**b**) cocultured with MDA-HER2, N87, or JIMT-1 HER2+ target cells at a 1:1 ratio. Following 24 h of culture, supernatant was harvested and analyzed for the presence of IFNγ and IL2 by ELISA. Empty media, NT T cells, HER2+ target cells cultured without the presence of effector cells, and the MDA cell line served as controls. Histograms represent the mean ± SD (*n* = 6, *OKT3-antiCD28/RPMI* vs. *OKT3-RetroNectin/LymphoONE*: * *p* < 0.05, ** *p* < 0.01).

Having established that *OKT3-antiCD28/RPMI*-generated CAR T cell products are enriched in effector memory (EM) T cells and *OKT3-RetroNectin/LymphoONE*-generated products in central memory (CM) T cells, we next investigated their effector function. First, we determined the production of interferon gamma (IFNγ) and interleukin-2 (IL-2) in the presence of immobilized HER2 target antigen by culturing EM- or CM-enriched HER2.CD28.z and HER2.41BB.z CAR T cells on plates coated with 1 μg/mL of immobilized HER2-Fc chimera protein. Media was collected after 24 h, and the concentration of IFNγ and IL-2 was determined by ELISA. Unmodified T cells served as controls. EM phenotype-enriched HER2.CD28.z and HER2.41BB.z CAR T cells produced more IFNγ and IL2 than their CM-enriched counterparts, although this only reached statistical significance for HER2.41BB.z CAR T cells (Figure 2a). No cytokine production was observed by unmodified T cells (NT) (IFNγ and IL-2, Figure 2a) or in the absence of the target antigen (data not presented), indicating that cytokine production is strictly dependent on the presence of the target antigen and expression of an antigen-specific CAR.

In parallel, we aimed to reveal whether the differentiation state has any impact on the exhaustion state of the CAR T cell products. T cell immunoglobulin and mucin-domain containing-3 (TIM-3), a transmembrane protein expressed on various effector cells, seems to be a reliable marker of T-cell exhaustion [29]. The amount of soluble TIM-3 produced by matrix metalloproteinases [30] has also been demonstrated to correlate with T exhaustion in various disorders [31]. Following 24 h incubation with immobilized HER2-Fc, CM phenotype-enriched HER2.CD28.z and HER2.41BB.z CAR T cells produced significantly higher amounts of sTIM-3 than their EM-enriched counterparts (Supplementary Figure S3), indicating the increased tendency for exhaustion of CM-enriched CAR T cell products.

Moreover, we investigated whether CM- or EM-directed differentiation has any impact on the activation profile of kinases and related proteins associated with T cell function. Thus, we performed a phospho-proteome profiling assay on CM- and EM-enriched CAR T cells to gain insight into the expression profile of signaling proteins upon short-term HER2-Fc stimulation. In both CD28.z CAR T products, phosphorylation of c-Jun N-terminal kinases (JNK) and proline-rich Akt substrate of 40 kDa (PRAS40) was increased compared to the 41BB.z products (Supplementary Figure S4). This is coherent with both JNK and PRAS40 acting downstream of CD28-mediated co-stimulation [32,33]. Notably, glycogen synthase kinase 3 (GSK3), a signaling molecule regulating T cell differentiation, has shown increased phosphorylation on S9 in CM-enriched CAR T cells products compared to EM-enriched products (Supplementary Figure S4), which mechanistically explains the delayed differentiation promoted by the *OKT3-RetroNectin/LymphoONE* protocol [34,35].

Next, we cocultured the various HER2-CAR T cell populations with HER2+ target cells (MDA-HER2, JIMT-1, or N87) at an effector to target (E:T) ratio of 1:1 to determine the IFNγ secretion. Unmodified T cells (NT) and HER2– MDA cells served as controls. In accordance with the previous results, all HER2-CAR T cell populations recognized HER2+ tumor cells and secreted a significant (*p* < 0.001) amount of IFNγ (MDA-HER2, JIMT-1, N87 panels, Figure 2b). Similar to HER2-Fc chimera stimulation, differentiated HER2.CD28.z CAR T cells produced greater amount of IFNγ than their less differentiated counterparts (Figure 2b). In contrast, HER2.41BB.z CAR T cell products enriched in a CM phenotype produced significantly more IFNγ than their EM-enriched counterparts (Figure 2b). There was no cytokine secretion in cocultures with unmodified T cells (NT) or HER2– MDA target cells, confirming specificity (Figure 2b).

### 3.3. Differentiated CAR T Cell Products Induce Stronger In Vitro Cytotoxicity

**Figure 3:**
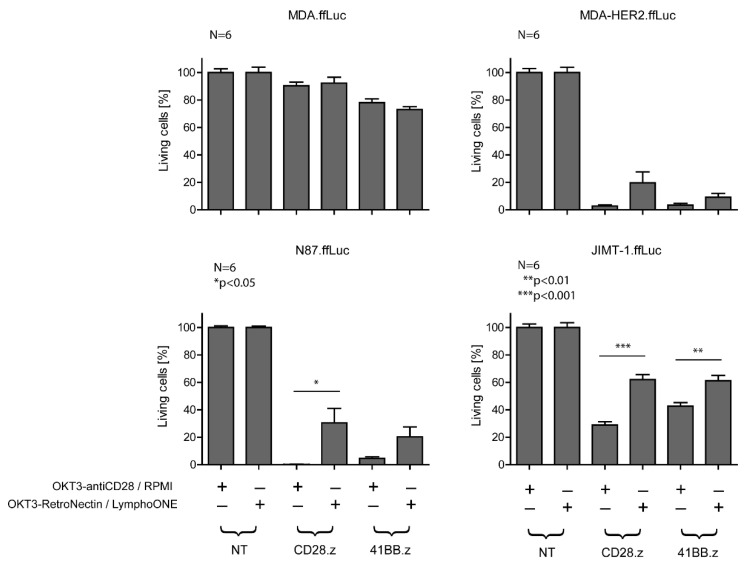
In vitro cytotoxicity of HER2-CAR T cells prepared with the two different stimulation protocols. The cytotoxic activity of CAR T cells was determined by a luciferase-based killing assay. MDA-HER2.ffLuc, N87.ffLuc, and JIMT-1.ffLuc were plated in 96-well flat-bottom plates at a concentration of 10^6^ cells/well in duplicates. After 4 h, 10^6^ CD28.z and 41BB.z HER2-CAR T cells prepared with the two different stimulation protocols were added. After 24 h, luciferase activity was determined. Empty media, NT T cells, HER2+ target cells cultured without the presence of effector cells, and the MDA.ffLuc cell line served as controls. Histograms represent the mean ± SD (*n* = 6, *OKT3-antiCD28/RPMI* vs. *OKT3-RetroNectin/LymphoONE*: * *p* < 0.05, ** *p* < 0.01, *** *p* < 0.001).

Next, we investigated the anti-tumor activity of HER2-CAR T cells in a firefly luciferase (ffLuc) activity-based cytotoxicity assay (Figure 3) at an E:T ratio of 1:1 using MDA-HER2.ffLuc, JIMT-1.ffLuc, and N87.ffLuc as target cells. MDA.ffLuc cells served as HER2– controls. Confirming the ELISA results, all types of HER2-CAR T cells recognized and killed HER2+ targets (Figure 3). There was no cytolytic activity in cocultures with unmodified T cells and MDA target cells (NT columns, MDA panel, Figure 3), indicating specificity. We confirmed that the increased amount of effector memory phenotypes in the *OKT3-antiCD28/RPMI*-stimulated CD28.z and 41BB.z CAR T cell products induced stronger CAR-specific cytotoxicity (MDA-HER2.ffLuc, JIMT-1.ffLuc, and N87.ffLuc, Figure 3) in comparison to the less differentiated CAR T cell products. This robust cytolytic effect was also exerted against the trastuzumab-resistant JIMT-1 cells. Nevertheless, this breast cancer cell line was the hardest target for effector cells based on its highest survival potential, and therefore we decided to perform further experiments on this cell line (JIMT-1.ffLuc, Figure 3).

### 3.4. Differentiated CAR T Cell Products Have a Greater Proliferative Capacity upon Repeated Stimuation In Vitro

**Figure 4:**
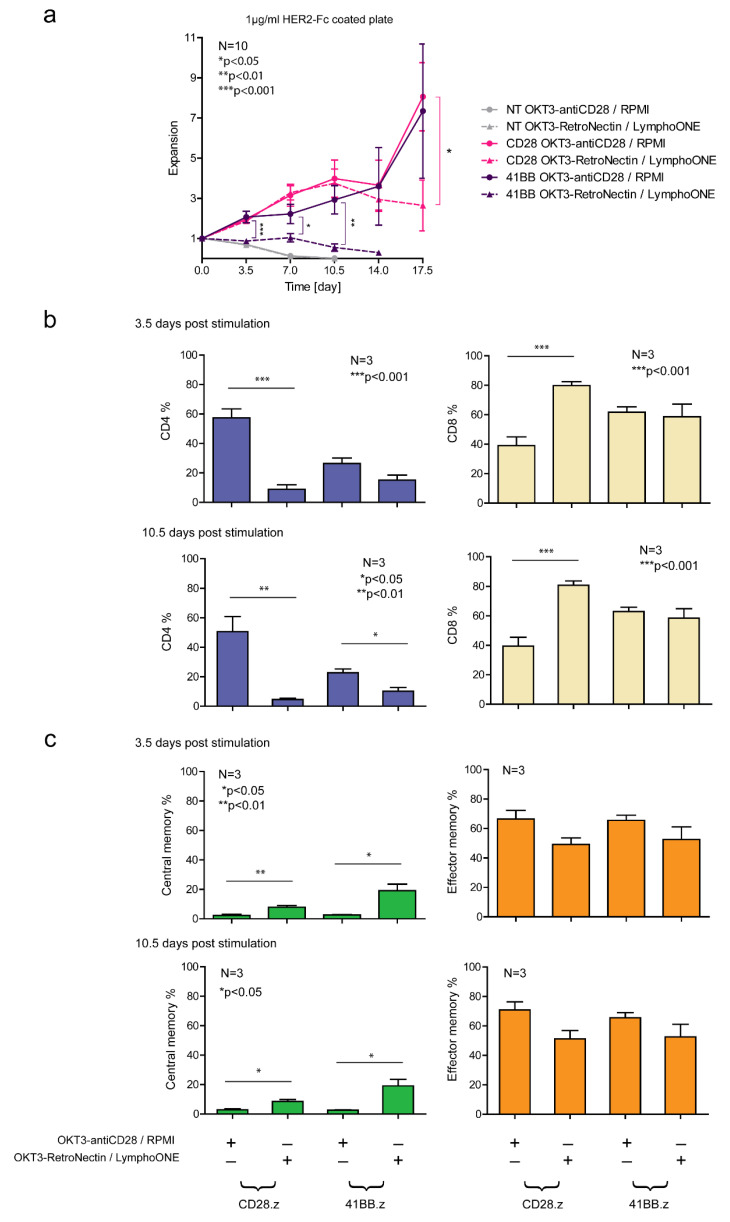
Phenotype distribution and persistence of antigen-stimulated HER2-CAR T cell products. Rechallenge experiment of NT and CAR T cell products prepared with two different stimulation and cell culture protocols. (**a**) NT or CAR T cells (2 × 10^5^) were placed onto 1 µg/mL HER2-Fc precoated plates in duplicates. Every 3.5 days, effector cell number was determined by flow cytometry. If it was available, the initial effector cell quantity was placed onto new plates in conditions identical to the beginning of the experiment. The expansion rate was calculated by dividing the total effector cell number on the present day by the effector cells plated in the beginning of the last 3.5- and 10.5-day round. The experiment was concluded for a subset if the expansion rate of the effector cells fell under 0.45. (**b**) CD4 and CD8 positivity was tested in the whole CAR T population by labeling with FITC-conjugated CD4 and Alexa Fluor 647-conjugated CD8 3.5 and 10.5 days after HER2-Fc stimulation. (**c**) Naïve (CCR7, CD45RA double-positive), central memory (CCR7-positive, CD45RA-negative), effector memory (CCR7, CD45RA double-negative), and terminal effector (CCR7-negative, CD45RA-positive) phenotypes were determined in the whole CAR T population by labeling with FITC-conjugated anti-human CCR7 and APC-conjugated anti-human CD45RA 3.5 and 10.5 days after HER2-Fc stimulation. Histograms of the naïve and the terminal effector phenotype frequency are provided in Supplementary Figure S2. Plotted values represent the mean ± SD (*n* = 3, *OKT3-antiCD28/RPMI* vs. *OKT3-RetroNectin/LymphoONE*: * *p* < 0.05, ** *p* < 0.01, *** *p* < 0.001).

Next, we aimed to investigate whether the differentiation state of the effector cells has any impact on their in vitro persistence. HER2-CAR T cells were bi-weekly restimulated on immobilized HER2-Fc (Figure 4a) molecules. NT T cells served as controls (Figure 4a). We found that in the presence of 1 µg/mL of immobilized HER2-Fc, CM-enriched HER2-CAR T cells had a reduced proliferative capacity in comparison to EM-enriched CAR T cell populations (CD28.z and 41BB.z solid lines vs. CD28.z and 41BB.z dashed lines, Figure 4a). In parallel, we characterized the effector and memory phenotype patterns of the rechallenged CAR T cells on days 3.5 and 10.5 of rechallenge. We found that for *OKT3-Retronectin/LymphoONE*-generated CAR T cell products, CAR-specific antigen stimulation reduced the frequency of CD4+ T cells (CD28.z and 41BB.z, *OKT3-RetroNectin/LymphoONE* columns, Figure 4b), and restrained a significant portion of CM T cells from EM-directed differentiation (CD28.z and 41BB.z, *OKT3-RetroNectin/LymphoONE* columns, Figure 4c). In contrast, *OKT-3-antiCD28/RPMI*-generated CAR T cell populations completely differentiated into effector memory and terminal effector T cells (CD28.z and 41BB.z, *OKT3-antiCD28/RPMI* columns, Figure 4c).

### 3.5. Effector Memory Phenotype-Enriched Car T Cell Products Induce Complete Tumor Eradication In Vivo

**Figure 5:**
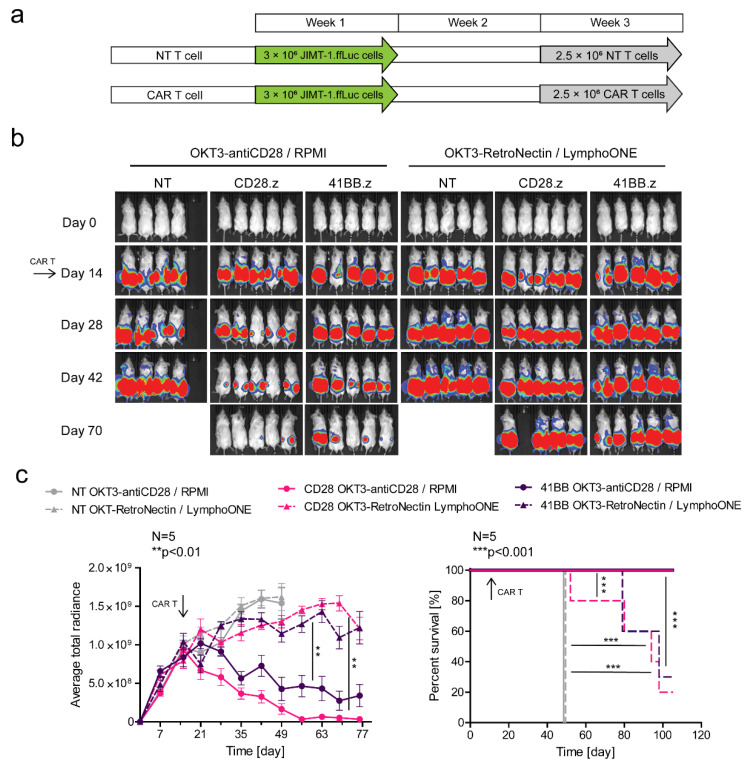
EM-enriched but not CM-enriched HER2-CAR T cells eliminate HER2-positive tumor xenografts in vivo. Mice were injected s.c. with 3 × 10^6^ JIMT-1.ffLuc cells. Mice on day 14 (arrow) received a single i.v. dose of 2.5 × 10^6^ NT T cells (NT *OKT3-antiCD28/RPMI* T cells, *n* = 4, grey, straight line; NT *OKT3-RetroNectin/LymphoONE* T cells, *n* = 5, grey, dashed line) or HER2-CAR T cells (CD28.z *OKT3-antiCD28/RPMI* T cells, *n* = 5, pink, straight line; CD28.z *OKT3-RetroNectin/LymphoONE* T cells, *n* = 5, pink, dashed line; 41BB.z *OKT3-antiCD28/RPMI* T cells, *n* = 5, purple, straight line; 41BB.z *OKT3-RetroNectin/LymphoONE* T cells, *n* = 5, purple, dashed line). Tumor growth was followed by bioluminescence imaging. (**a**) Outline of the treatment schedule. (**b**) Representative images of JIMT-1.ffLuc-injected animals. (**c**, left panel) Quantitative bioluminescence imaging data of JIMT-1.ffLuc xenografts (average total radiance = photons/s/cm^2^/sr; *OKT3-antiCD28/RPMI* HER2-CAR vs. *OKT3-RetroNectin/LymphoONE* HER2-CAR treatment: ** *p* < 0.01). (**c**, right panel) Kaplan-Meier survival curve (*OKT3-antiCD28/RPMI* HER2-CAR vs. *OKT3-RetroNectin/LymphoONE* HER2-CAR treatment: *** *p* < 0.001; *OKT3-RetroNectin/LymphoONE* NT vs. *OKT3-RetroNectin/LymphoONE* HER2-CAR treatment: *** *p* < 0.001). Histograms represent mean ± SD.

In the final set of experiments, we focused on comparing the anti-tumor activity and expansion/persistence of EM- and CM-enriched CAR T cell products in vivo using a subcutaneous (s.c.) HER2+ JIMT-1 xenograft model, in which either tumor cells or CAR T cells were genetically modified to express firefly luciferase (ffLuc). We injected 3 × 10^6^ JIMT-1.ffLuc tumor cells s.c. on both flanks on day 0, and then a single i.v. dose of 2.5 × 10^6^ HER2-CAR T cells on day 14 post-tumor cell injection. Tumor progression was monitored by an IVIS^®^ Spectrum In Vivo Imaging System (Perkin Elmer) (Figure 5a,b). EM-enriched CD28.z and 41BB.z CAR T cells had greater anti-tumor activity than their CM-enriched counterparts, resulting in an improved overall survival advantage (Figure 5b,c). For EM-enriched CAR T cell products, CD28.z CAR T cells had greater antitumor activity than 41BB.z CAR T cells (Figure 5b,c), mirroring our in vitro studies. NT T cells had no anti-tumor activity, confirming specificity (Figure 5b,c).

### 3.6. Effector Memory Phenotype-Enriched Car T Cell Products Show Faster Expansion In Vivo

**Figure 6:**
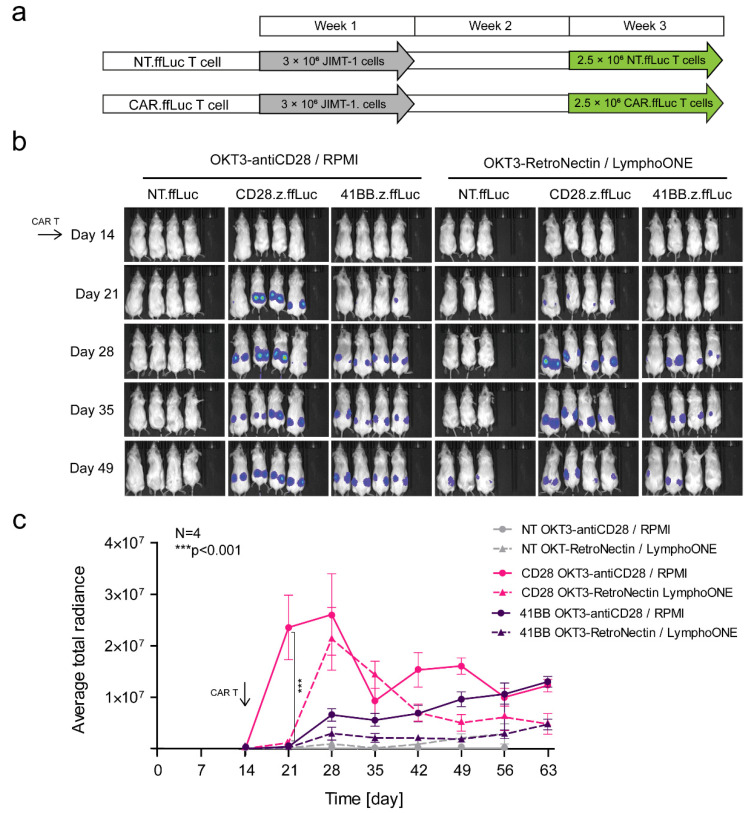
EM-enriched CD28.z CAR.ffLuc T cells expand within one week post-infusion at tumor xenograft sites in vivo. Mice were injected s.c. with 3 × 10^6^ JIMT-1 cells. Mice on day 14 (arrow) received a single i.v. dose of 2.5 × 10^6^ NT.ffLuc T cells (NT.ffLuc *OKT3-antiCD28/RPMI* T cells, *n* = 4, grey, straight line; NT.ffLuc *OKT3-RetroNectin/LymphoONE* T cells, *n* = 3, grey, dashed line) or a single i.v. dose of 5 × 10^6^ HER2-CAR.ffLuc T cells (CD28.z.ffLuc *OKT3-antiCD28/RPMI* T cells, *n* = 4, pink, straight line; CD28.z.ffLuc *OKT3-RetroNectin/LymphoONE* T cells, *n* = 4, pink, dashed line; 41BB.z.ffLuc *OKT3-antiCD28/RPMI* T cells, *n* = 4, purple, straight line; 41BB.z.ffLuc *OKT3-RetroNectin/LymphoONE* T cells, *n* = 4, purple, dashed line). Tumor infiltration was followed by bioluminescence imaging. (**a**) Outline of the treatment schedule. (**b**) Representative images of JIMT-1-injected animals. (**c**) Quantitative bioluminescence imaging data of HER2-CAR.ffLuc T cells infiltrating JIMT-1 xenografts (average total radiance = photons/s/cm^2^/sr; CD28.z.ffLuc *OKT3-antiCD28/RPMI* HER2-CAR vs. CD28.ffLuc *OKT3-RetroNectin/LymphoONE* HER2-CAR.ffLuc: *** *p* < 0.001) Histograms represent mean ± SD.

Having established that EM-enriched CAR T cell products had greater anti-tumor activity in vivo, we wanted to determine if this correlates with greater expansion and/or persistence post-infusion. We performed the same in vivo experiments as described above with the only difference being that this time, CAR T cells expressed ffLuc (NT.ffLuc, CD28.z.ffLuc, 41BB.z.ffLuc) and not the JIMT-1 tumor cells (Figure 6b). EM-enriched CD28.z CAR T cells expanded faster and for a longer time period than CM-enriched T cell products (*OKT3-anti-CD28/RPMI* CD28.z.ffLuc vs. *OKT3-RetroNectin/LymphoONE* CD28.z.ffLuc, *p* < 0.01, Figure 6b,c). In accordance with this result, EM-enriched 41BB.z CAR T cells also outperformed their less differentiated counterparts (*OKT3-anti-CD28/RPMI* 41BB.z.ffLuc vs. *OKT3-RetroNectin/LymphoONE* 41BB.z.ffLuc, Figure 6b,c). 41BB.z CAR T cells expanded slowly post-infusion for the entire duration of the experiment, whereas CD28.z CAR T cells contracted after their initial expansion. NT.ffLuc T cells did not expand, in coherence with having no anti-tumor activity (Figure 6b,c).

## 4. Discussion

The optimal composition of CAR T cell products for solid tumors remains elusive. To address this issue, we have performed a systematic in vitro and in vivo comparison of the effector function of less differentiated (CM-enriched) and more differentiated (EM-enriched) HER2.CD28.z and HER2.41BB.z CAR T cell products. We demonstrated here that EM-enriched HER2-CAR T cells have superior effector function in vitro, including better clonal expansion in a repeated stimulation assay. This translates to improved effector function, as judged by antitumor activity and ability to expand in vivo. In addition, CD28.z. CAR T cells outperform 41BB.z CAR T cells in cytolytic activity as well as in in vivo antitumor activity and expansion.

In the past decade, IL-2 was used as a gold standard cytokine for cultivation of CAR T cells [36]. However, recent preclinical studies have shown that other gamma-chain cytokines (such as IL-7 and IL-15) promote a less-differentiated T cell phenotype, resulting in longer persistence and better anti-tumor effects in comparison to IL-2 [12]. Moreover, IL-2 induces Fas-mediated T-cell apoptosis that has a negative impact on long-term cytotoxic effects [37]. Thus, we decided to activate and maintain our CAR T cells in an IL-7/IL-15-supplemented cell culture media. On the other hand, IL-2, as a clinically approved and easily accessible molecule on the market, has relatively low cost in contrast to IL-7/IL-15. Thus, since expensive production is a major barrier to the spread of CAR T cell therapies, it remains to be seen if the superior anti-tumor effect of well-differentiated CAR T cells in solid tumors could also be achieved using IL-2-based expansion, in spite of the tendency of IL-2-stimulated cells for early apoptosis.

For our comparison, we generated CAR T cells with two different protocols and demonstrated that *OKT3-RetroNectin/LymphoONE*-stimulated CD28.z and 41BB.z CAR T cell products contained a significantly higher amount of less-differentiated central memory T cells and CD8+ cytotoxic lymphocytes. This finding is reinforced by the enhanced phosphorylation of GSK3 in these subsets, which through inhibition of GSK3 was shown to arrest effector T cell differentiation and to generate CD8+ memory stem cells [34,35]. It is also consistent with a previous study in which Gargett et al. [17] reported that OKT3/RetroNectin treatment with IL-2-supplemented RPMI culture medium induced higher amounts of the CD45RA+ stem/memory subset and dramatically increased the proportion of CD8+ T cells. Furthermore, our data support the findings of Stock et al. [18], suggesting that RetroNectin-based activation results in an enrichment of CD8+ cytotoxic and less-differentiated naïve-like (CD45RA+CCR7+) CAR T cells when using a third-generation CAR targeting CD19.

With our functional in vitro studies, we demonstrated that in the presence of immobilized target antigens, CAR T products rich in effector memory T cells secreted higher amounts of IFNγ and IL-2, and induced more potent CAR-specific anti-tumor activity. Additionally, these cells produced significantly lower amounts of soluble TIM-3, suggestive of their lower exhaustion level [29,30,31]. Furthermore, these well-differentiated CAR T cell products expanded better in rechallenge assays on immobilized target antigens. However, less-differentiated 41BB.z CAR T cells secreted higher amount of IFNγ when HER2+ was present on cellular targets. This finding might be partially explained by the multidirectional modulatory effects of tumor cells on the cytokine secretion of T cell subsets [38], warranting additional studies on this aspect.

Since differentiated, EM-enriched CAR T cell products exhibited stronger cytotoxicity and better expansion in rechallenge assays, we performed in vivo experiments to investigate whether the different phenotypical characteristics of CAR T cell products influence anti-tumor activity. We found that, similarly to the in vitro results, CAR T cell products rich in EM T cells expanded faster in vivo, and yielded complete tumor eradication, as opposed to the less differentiated, CM-enriched products. Our findings are in contrast to studies that have been conducted targeting CD19 with CAR T cells. For example, pre-clinical and clinical leukemia studies have demonstrated the superior anti-tumor activity and long-term persistence of less-differentiated CD19-CAR T cell products with a high frequency of naïve and central memory T cell subsets [12,13,14,15]. This could be explained by several factors, including the targeted antigen [39] or the mere fact that the molecular composition of the cell surface of solid tumors and leukemia cells differs [40]. In addition, leukemic blasts are readily accessible and relatively defenseless to circulating CAR T cells, which is in stark contrast to the complex tumor microenvironment (TME) of solid tumors. The latter contains a dense extracellular matrix, which poses physical obstacles [28,41,42,43], as well as cancer-associated fibroblasts (CAFs) [44], tumor-infiltrating macrophages [45], and regulatory T and B cells that are potential negative immune regulators [46]. Thus, it appears that the decreased cytolytic activity of CM-enriched CAR T cells is sufficient for the eradication of leukemic blasts, but insufficient to overcome the immunosuppressive TME of solid tumors, which requires a stronger T cell activation. One way to prove this is by incorporating a CD28 rather than a 41BB costimulatory domain into the CAR construct. Indeed, other groups have shown that CD28 co-stimulation results in greater T cell activation in comparison to 41BB co-stimulation in the context of solid tumor-specific CAR T cells [47]. Limited 41BB.z CAR T cell activation could potentially also be overcome by including a second zeta domain with the CAR [48]. Finally, additional genetic modifications, including the transgenic expression of cytokines and/or deletion of negative regulators, are attractive strategies to further enhance the anti-tumor activity of CD28.z or 41BB.z CAR T cells [49]. Based on our studies, it seems advisable to explore their benefits in EM- and CM-enriched CAR T cell populations.

Our in vitro data showed that in the *OKT3-antiCD28/RPMI*-stimulated CD28.z CAR T cell products, the initial 60%/40% CD4+/CD8+ ratio is preserved after repeated stimulation. In contrast, in the OKT3-RetroNectin-activated CD28.z CAR T cell products, the percentage of CD4+ cells decreased from ~50% to below 10% during the same period of HER2 challenge. In the case of 41BB.z, the antigen challenge reduced the original ~55% CD4+ population by half, even with the classical expansion protocol, and using RetroNectin/LymphoONE stimulation further reduced this cell subset to below 20%. In general, CD8+ T cells are considered as mediators of direct tumor cell eradication, while CD4+ T helper lymphocytes, among numerous other functions, facilitate the activation and proliferation of CD8+ T cells. However, for CAR T cells, CD4+ T cells possess direct anti-tumor activity comparable to cytotoxic CD8^+^ CAR T cells [50,51]. It was reported that CD4+ helper and CD8+ cytotoxic T cells exert a synergistic antitumor effect in a 1:1 ratio, positively influencing tumor eradication [52], and achieve high remission rates during the treatment of B-ALL patients with CD19 CAR-T cells [15]. Recently, it was also shown that TH9, but not TH2, differentiation of CD4+ CAR T cells promotes the function of CD8^+^ CAR T cells in the tumor environment [53]. Thus, our and other investigators’ findings support the concept that maintaining a balanced composition of CD8+ and CD4+ CAR T cells is advantageous when targeting solid tumors [54].

## 5. Conclusions

In conclusion, we have optimized culture conditions for producing CAR T cell populations that are enriched in central memory or effector memory T cell subsets. As a model system, we used HER2-CAR T cells with either CD28 or 41BB costimulatory domains and HER2+ tumor cells. Our results demonstrated that effector memory-enriched CAR T cell products with a balanced composition of CD8+ and CD4+ T cells perform best, both in vitro and in in vivo preclinical models. Thus, CAR T cell generation protocols have a significant impact on the potency of the resulting CAR T cells. Importantly, our results suggest that ‘one size does not fit all’, and that CAR design and culture conditions need to be fine-tuned for the intended clinical application.

## Data Availability

The data presented in this study are available in this article and its Supplementary Materials.

## Supplementary Materials

The following are available online at https://www.mdpi.com/article/10.3390/cancers13174301/s1, Figure S1: Assessing the memory phenotype distribution of -CAR T cells: flow cytometry gating strategy. Figure S2: Generation of 12 different T cell products with two distinct cell culturing and two distinct stimulating methods using HER2.CD28.z or HER2.41BB.z CAR constructs and non-transduced (NT) controls: distribution of expression levels, % CAR positivity, CD4+/CD8+ and memory phenotypes. Figure S3: Relative TIM-3 secretion of FRP5-derived HER2-CAR T cells following HER2-stimulation. Figure S4: Hierarchical clustering of phosphoprotein levels upon HER2 stimulation of HER2-CAR T cell samples prepared with distinct cell culturing and stimulating methods.
