# Supplementary material for: Cytolytic Activity of CAR T Cells and Maintenance of Their CD4+ Subset Is Critical for Optimal Antitumor Activity in Preclinical Solid Tumor Models
Source: Cancers (Basel). 2021 Aug 26;13(17):4301. doi: 10.3390/cancers13174301 (PMC8428348; doi:10.3390/cancers13174301)
Supplement: Supplementary file 1 [file cancers-13-04301-s001.zip › cancers-1326526-supplementary_v5_final.pdf]

Supplementary Materials

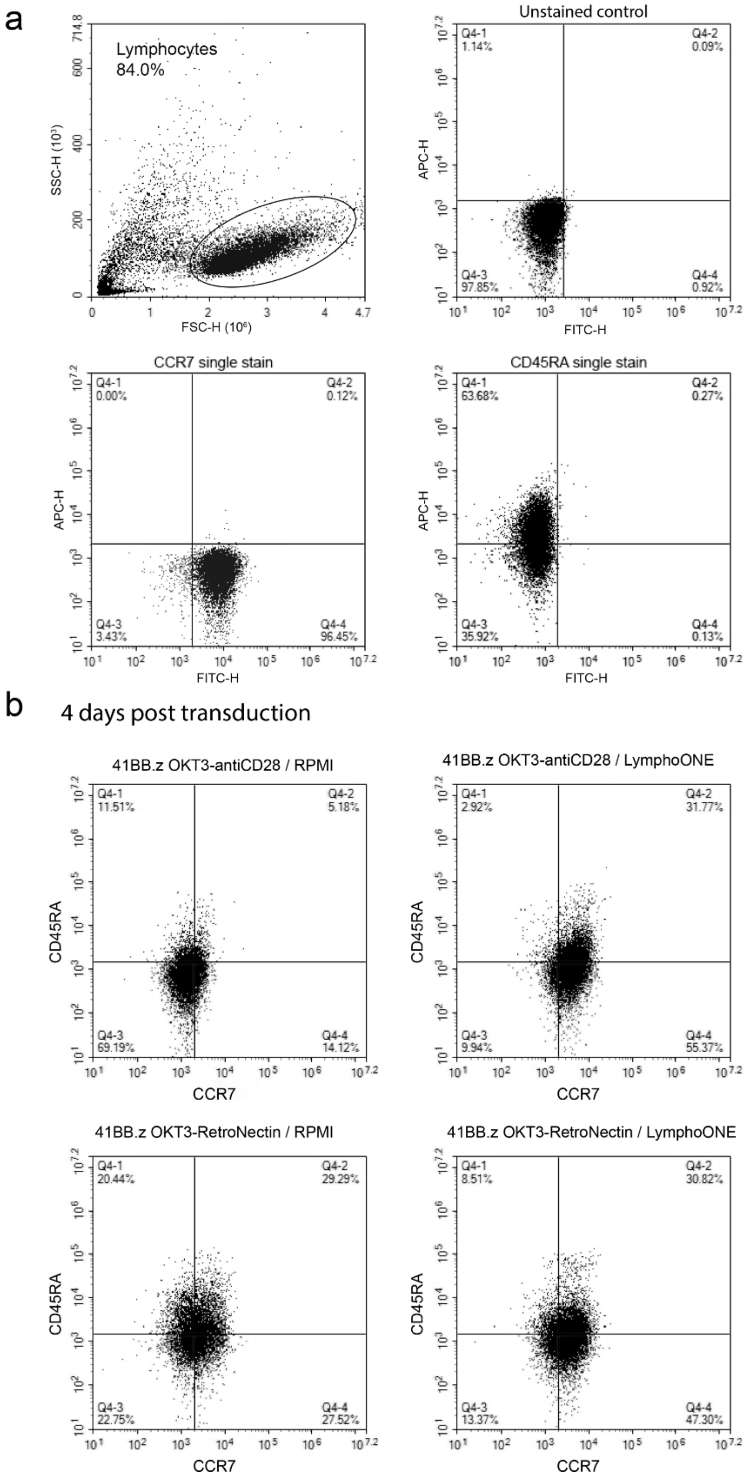

Figure S1. : Assessing the memory phenotype distribution of CAR T cells: flow cytometry gating strategy. Naïve (CCR7, CD45RA double positive), central memory (CCR7 positive, CD45RA negative) effector memory (CCR7, CD45RA double negative) and terminal effector (CCR7 negative, CD45RA positive) phenotype was determined by labelling with FITC conjugated anti-human CCR7 and APC conjugated anti-human CD45RA 4 days post transduction.

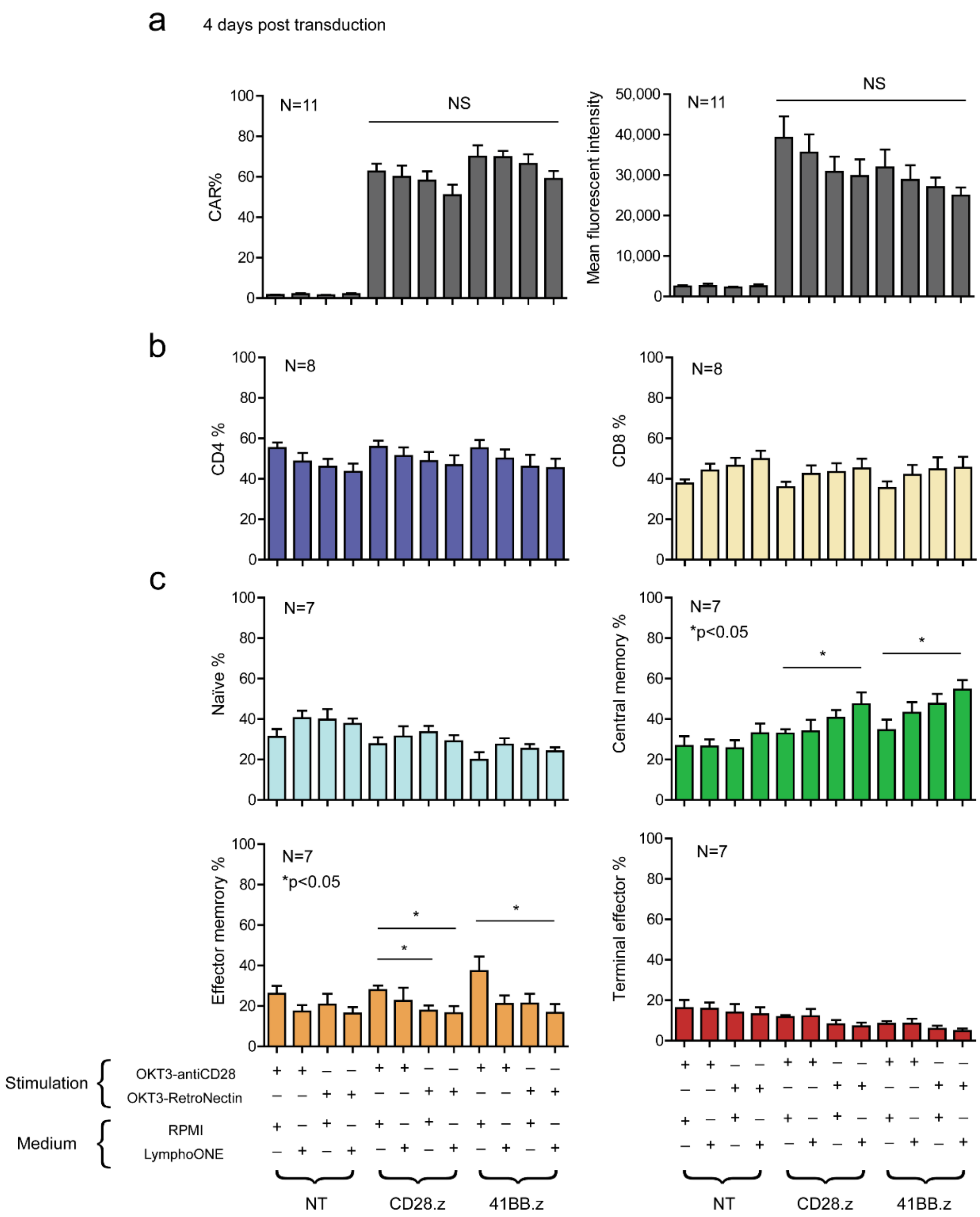

Figure S2. : Generation of 12 different T cell products with two distinct cell culturing and two distinct stimulating methods using HER2.CD28.z or HER2.41BB.z CAR constructs and non-transduced (NT) controls: distribution of expression levels, % CAR positivity, CD4+/CD8+ and memory phenotypes. (a) HER2-CAR expression quantified using a HER2-Fc fusion protein followed by Alexa Fluor 647 conjugated anti-human IgG staining (N=11). (b) CD4 and CD8 positivity tested by labelling with FITC conjugated anti-CD4 and Alexa Fluor 647 conjugated anti-CD8 4 days post transduction (N=8). (c) Naïve (CCR7, CD45RA double positive), central memory (CCR7 positive, CD45RA negative) effector memory (CCR7, CD45RA double negative) and terminal effector (CCR7 negative, CD45RA positive) phenotype determined by labelling with FITC conjugated anti-human CCR7 and APC conjugated anti-human CD45RA 4 days post transduction (N=7). Histograms represent the mean ± SD (*$p < 0.05$).

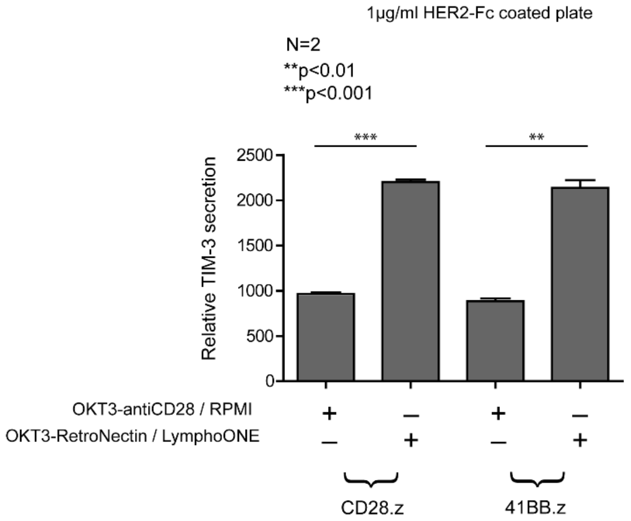

Figure S3. : Relative TIM-3 secretion of FRP5-derived HER2-CAR T cells following HER2-stimulation. 1 × 10^6/ml CAR T cells were incubated on 1µg/ml HER2-Fc precoated plates for 24 hours on 37°C. Following 24 hours of culture, supernatant was harvested and analyzed for the presence of soluble TIM-3 by membrane-based sandwich immunoassay. Histograms representthe mean ± SD (**$p < 0.01$; ***$p < 0.001$).

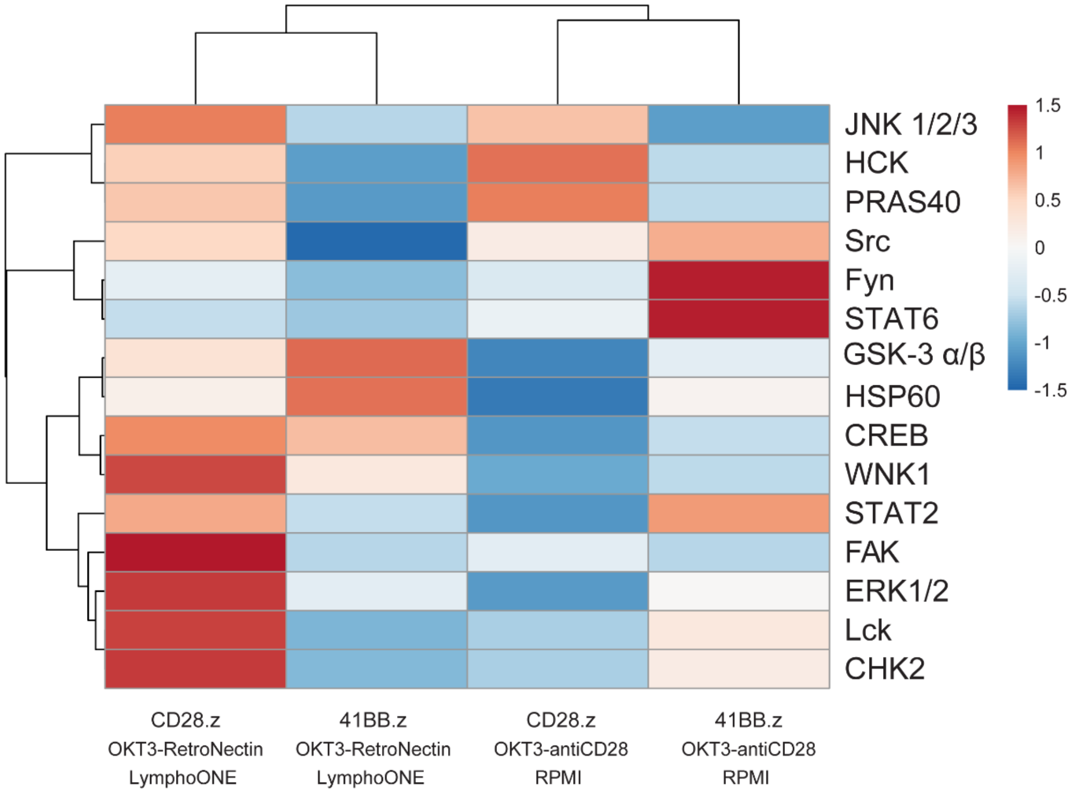

Figure S4. : Hierarchical clustering of phosphoprotein levels upon HER2 stimulation of HER2-CAR T cell samples prepared with distinct cell culturing and stimulating methods. CAR T cells were incubated on 1µg/ml HER2-Fc precoated plates for 0.5, 2.5 or 7 minutes on 37°C. Samples from all endpoints were pooled to cover all major changes during the initial downstream signaling phase and measured using a Proteome Profiler Human Phospho-Kinase Array kit. Unsupervised two-way hierarchical clustering on the average kinase phosphorylation values using correlation distance and average linkage was performed using ClustVis.
